# Roles of preoperative C-reactive protein are more relevant in buccal cancer than other subsites

**DOI:** 10.1186/s12957-017-1116-5

**Published:** 2017-02-16

**Authors:** Shiao Fwu Tai, Huei-Tzu Chien, Chi-Kuang Young, Chung-Kang Tsao, Alba de Pablo, Kang-Hsing Fan, Chun-Ta Liao, Hung-Ming Wang, Chung-Jan Kang, Joseph Tung-Chieh Chang, Shiang-Fu Huang

**Affiliations:** 1 Department of Otolaryngology, Chang Gung Memorial Hospital, Linkou, Taiwan; 2 Chang Gung University, Taoyuan, Taiwan, Republic of China; 3 Department of Public Health, Chang Gung University, Taoyuan, Taiwan, Republic of China; 4 Department of Otolaryngology, Chang Gung Memorial Hospital, Keelung, Taiwan, Republic of China; 5 Plastic and Reconstructive Surgery, Chang Gung Memorial Hospital, Linkou, Taiwan, Republic of China; 6 Department of Radiation Oncology, Chang Gung Memorial Hospital, Chang Gung University, Taoyuan, Taiwan, Republic of China; 7 Department of Medical Oncology, Chang Gung Memorial Hospital, Chang Gung University, Taoyuan, Taiwan, Republic of China

**Keywords:** Oral squamous cell carcinoma, C-reactive protein, Prognosticator

## Abstract

**Background:**

C-reactive protein (CRP) is an early marker for inflammation, and a relationship between serum CRP levels and survival in oral cancer has been demonstrated previously. In this study, we investigated the roles of CRP in different oral cancer subsites.

**Methods:**

Three hundred and forty-three oral squamous cell carcinoma patients between June 1999 and March 2015 were retrospectively reviewed. Serum CRP levels were measured preoperatively.

**Results:**

The elevation of CRP levels (≥5.0 mg/L) was significantly correlated with pathologic tumor status, pathologic nodal status, nodal extracapsular spread, tumor stage, skin invasion, tumor depth (≥10 mm), and bone invasion. The correlation between elevation of CRP and clinicopathologic factors was more evident in the buccal cancer compared to other tumor subsites. The disease-free survival and overall survival correlation was significant in buccal cancer (*p* = 0.003 and *p* < 0.001) but not in tongue cancer (*p* = 0.119 and *p* = 0.341) or other oral cancer subsites (*p* = 0.246 and *p* = 0.696).

**Conclusions:**

Preoperative serum CRP level was a prognosticator in oral squamous cell carcinoma, and its effect was more prominent in buccal cancer that occurs more frequently in areca-quid (AQ) endemic regions.

**Electronic supplementary material:**

The online version of this article (doi:10.1186/s12957-017-1116-5) contains supplementary material, which is available to authorized users.

## Background

Oral cavity cancer is a malignancy with increased incidence in recent years. As it is widely known, alcohol, betel nut, and cigarette consumption increased the risks of oral cavity cancer [1, 2]. Chronic exposure to these carcinogenic factors can cause transform the oral cavity mucosa into malignancy. Part of the Taiwanese population commonly consumes cigarettes and betel nuts; it makes the oral cancer fifth in the top ten common cancers in Taiwan, and its incidence still increases in recent years [3]. For oral cancer treatments, a decision of surgical intervention, radiotherapy, chemotherapy, or combination, depends on cancer staging, lymph node metastasis, pathologic factors, and distant metastasis.

**Fig. 1:**
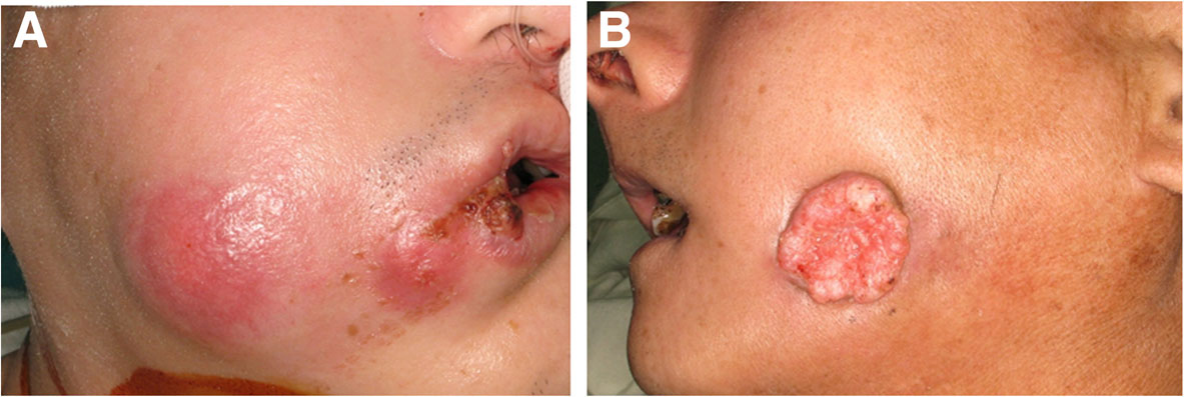
Patients with same tumor invasion into the skin. **a** Combined with peri-tumoral inflammation. **b** Without peri-tumoral inflammation

In recent years, more and more research studies proved that in addition to preoperative cancer staging, the patients’ preoperative condition plays an important role in predicting the prognosis of oral cavity cancer. Inflammation, which may contribute to the formation of oral cavity cancer or was resulted from the host reaction to the tumor progression (Fig. 1), was also found to correlate with patient’s prognosis [4]. Some inflammatory markers such as interleukin-6, tumor necrotic factor, and C-reactive protein (CRP) were recently identified as prognostic markers in oral cavity cancer [4–7]. CRP is an acute phase protein, which is synthesized by the liver and released into the bloodstream within several hours after tissue injury, being able to reflect infection or an inflammatory status [8]. In many human cancers, CRP has a role as a prognostic predicting factor [5, 7, 9–11].

We previously demonstrated that CRP was an independent prognostic factor in oral cavity squamous cell carcinoma (OSCC) [7]. However, the studies were limited by the case number and follow-up period. In this study, we expanded our patient number and included a longer follow-up period to clarify the prognostic role in OSCC. Also, we stratified our patients according to different tumor subsites. We hope that the analysis in this study will clarify the true prognostic value of CRP in OSCC.

## Methods

### OSCC patients

Three hundred and forty-three patients between June 1999 and March 2015 in Chang Gung Memorial Hospital were retrospectively recruited. The inclusion criteria were primary OSCC without previous treatment before. The exclusion criteria were patients with verrucous carcinoma or distant metastasis. All patients included in this study received radical surgery in curative intent and with or without adjuvant chemo-radiation therapy. After treatments, all the patients were followed up regularly in the clinic and ended on September 2015 or on the date that the patients expired.

### OSCC staging and treatment

The patients in this series underwent an extensive preoperative survey, which included a detailed medical history and a complete physical examination, complete blood count, routine blood biochemistry, chest radiographs, computed tomography or magnetic resonance imaging scans of the head and neck, bone scan or positron emission tomography (PET), and liver ultrasound. The tumor staging followed the guidelines of the American Joint Committee on Cancer (7th edition) [12]. The tumor excisions in all patients were done by ≥1 cm safety margin. The tumor invasiveness parameters, which included tumor size, tumor cell differentiation, lymph node metastasis, lymph node extracapsular spread (ECS), depth of tumor invasion, perineural invasion, and soft tissue and lymphovascular invasion, were documented in the pathology report. For patients with advanced tumor stage (T3 or T4), lymph node ECS, tumor depth ≥10 mm, or poor differentiation, postoperative radiotherapy or concomitant chemoradiotherapy would be suggested [13].

### Measurement of CRP

The serum CRP level was measured preoperatively at the time of tissue diagnosis. It was measured before any medical treatment was delivered, including antibiotics [6, 13]. The levels of CRPs were measured by an auto-analyzer (Hitachi Medico, Tokyo, Japan). Elevation of serum CRP level was defined at a cut point of ≥5.0 mg/L.

### Statistical analysis

The mean values of preoperative CRP in different tumor subistes were compared using ANOVA. Chi-square test with univariate analysis were used in this study. Survival differences were compared with the log-rank test. SPSS software, version 18.0 (SPSS, Chicago, IL, USA), was used for data analysis. A two-sided *p* value ≤0.05 was defined as statistically significant.

### Ethics approval and consent to participate

All patients signed informed consent for participation of this study. This study had ethics approval and consent by the ethic committee in Chang Gung Memorial Hospital (Institutional Review Board of Chang Gung Medical Foundation, IRB No. 201600949B0), Taiwan, Republic of China on August 24, 2016.

## Results

### Patient characteristics

The clinical information of 343 OSCC patients in this study were shown in Table 1 (318 men and 25 women) and in Additional file 1. Tongue (*n* = 132, 38.5%) and buccal mucosa (*n* = 126, 36.7%) were the most common OSCC sites in this study. Pathological tumor stage distributed as stage I (*n* = 76, 22.2%), stage II (*n* = 66, 19.2%), stage III (*n* = 43, 12.5%), stage IVa (*n* = 133, 38.8%), and stage IVb (*n* = 25, 7.3%). The mean preoperative CRP was 6.96 mg/L (standard deviation (SD) ±12.06). The tumor stages were not different between different tumor subsites (*p* = 0.100). The mean CRP level was 5.90 mg/L (±SD 10.53), 8.37 mg/L (±SD 15.01), and 6.52 mg/L (±SD 8.90) in tongue cancer, buccal cancer, and other oral cavity cancers, respectively. The CRP levels were not significantly different between subsites (ANOVA *p* = 0.240).

**Table 1:** Characteristics of 343 patients with OSCC

Characteristic	Value
Age (year)	
Mean (±SD)	52.21 (±11.00)
Range	27.0–84.0
Gender	
Male	25 (7.3%)
Female	318 (92.7%)
Site of primary cancer	
Tongue	132 (38.5%)
Mouth floor	12 (3.5%)
Lip	24 (7.0%)
Buccal mucosa	126 (36.7%)
Alveolar ridge	31 (9.0%)
Hard palate	2 (0.6%)
Retromolar trigone	16 (4.7%)
Pathologic tumor status	
1	91 (26.5%)
2	119 (34.7%)
3	24 (7.0%)
4a	85 (24.8%)
4b	24 (7.0%)
Pathologic N stage	
N0	201 (58.6%)
N1	47 (13.7%)
N2b	79 (23.0%)
N2c	15 (4.4%)
N3	1 (0.3%)
Pathologic stage	
I	76 (22.2%)
II	66 (19.2%)
III	43 (12.5%)
IVa	133 (38.8%)
IVb	25 (7.3%)

### Association between CRP level with clinicopathologic parameters and prognosis

The relationship between CRP levels and tumor clinicopathologic parameters in OSCCs were examined and showed in Table 2. Advanced tumor status (*p* < 0.001), tumor stage (*p* < 0.001), skin invasion (*p* < 0.001), bone invasion (*p* < 0.001), and tumor depth ≥10 mm (*p* < 0.001) and advanced pathologic nodal status (*p* = 0.006) and lymphatic invasion (*p* = 0.068) were significantly correlated with CRP elevation (CRP ≥ 5.0 mg/L).

**Table 2:** Association between CRP and clinicopathologic parameters (*n* = 343)

Characteristic	CRP				*p* value
	Negative		Positive		
Pathologic tumor status					
Early (T1–T2) (*n* = 210)	181	(86.2%)	29	(13.8%)	*<0.001*
Advanced (T3–T4) (*n* = 133)	68	(51.1%)	65	(48.9%)	
Pathologic N stage					
N0 (*n* = 201)	156	(77.6%)	45	(22.4%)	*0.006*
N1 (*n* = 47)	36	(76.6%)	11	(23.4%)	*0.002* ^a^
N2 (*n* = 94)	57	(60.6%)	37	(39.4%)	
N3 (*n* = 1)	0	(0.0%)	1	(100.0%)	
Nodal status					
(−) metastasis, (−) ECS (*n* = 201)	156	(77.6%)	45	(22.4%)	*0.006*
(+) metastasis, (−) ECS (*n* = 59)	44	(74.6%)	15	(25.4%)	*0.002* ^a^
(+) metastasis, (+) ECS (*n* = 83)	49	(59.0%)	34	(41.0%)	
Differentiation					
Well (*n* = 107)	77	(72.0%)	30	(28.0%)	0.308
Moderate (*n* = 192)	144	(75.0%)	48	(25.0%)	0.538^a^
Poor (*n* = 44)	28	(63.6%)	16	(36.4%)	
Tumor stage					
Early (I–II) (*n* = 142)	123	(86.6%)	19	(13.4%)	*<0.001*
Advanced (III–IV) (*n* = 201)	126	(62.7%)	75	(37.3%)	
Skin invasion					
No (*n* = 304)	231	(76.0%)	73	(24.0%)	*<0.001*
Yes (*n* = 39)	18	(46.2%)	21	(53.8%)	
Nerve invasion					
No (*n* = 229)	171	(74.7%)	58	(25.3%)	0.221
Yes (*n* = 114)	78	(68.4%)	36	(31.6%)	
Blood vessel invasion					
No (*n* = 331)	242	(73.1%)	89	(26.9%)	0.322^b^
Yes (*n* = 12)	7	(58.3%)	5	(41.7%)	
Lymphatic invasion					
No (*n* = 334)	245	(73.4%)	89	(26.6%)	0.068^b^
Yes (*n* = 9)	4	(44.4%)	5	(55.6%)	
Tumor depth (≥10 mm)					
No (*n* = 160)	137	(85.6%)	23	(14.4%)	*<0.001*
Yes (*n* = 183)	112	(61.2%)	71	(38.8%)	
Bone invasion					
No (*n* = 275)	215	(78.2%)	60	(21.8%)	*<0.001*
Yes (*n* = 68)	34	(50.0%)	34	(50.0%)	

*ECS* extracapsular spread

^a^χ^2^ trend test

^b^Fisher’s exact test

We further analyzed the association between CRP and clinicopathologic factors in different tumor sites (buccal, tongue, and other locations). In buccal cancer (Table 3), CRP elevation (CRP ≥ 5.0 mg/L) was significantly associated with advanced tumor status (*p* < 0.001), advanced stage (*p* = 0.001), advanced pathological nodal status (*p* = 0.009), nodal status with ECS (*p* = 0.003), tumor depth ≥ 10 mm (*p* < 0.001), skin invasion (*p* < 0.001), nerve invasion (*p* = 0.042), and bone invasion (*p* = 0.011).

**Table 3:** Association between CRP and clinicopathologic parameters in buccal cancer (*n* = 126)

Characteristic	CRP				*p* value
	Negative		Positive		
Pathologic tumor status					
Early (T1–T2) (*n* = 71)	60	(84.5%)	11	(15.5%)	*<0.001*
Advanced (T3–T4) (*n* = 55)	25	(45.5%)	30	(54.5%)	
Pathologic N stage					
N0 (*n* = 70)	52	(74.3%)	18	(25.7%)	*0.009*
N1 (*n* = 18)	15	(83.3%)	3	(16.7%)	*0.006* ^a^
N2 (*n* = 37)	18	(48.6%)	19	(51.4%)	
N3 (*n* = 1)	0	(0.0%)	1	(100.0%)	
Nodal status					
(−) metastasis, (−) ECS (*n* = 70)	52	(74.3%)	18	(25.7%)	*0.003*
(+) metastasis, (−) ECS (*n* = 27)	21	(77.8%)	6	(22.2%)	*0.005* ^a^
(+) metastasis, (+) ECS (*n* = 29)	12	(41.4%)	17	(58.6%)	
Differentiation					
Well (*n* = 48)	28	(58.3%)	20	(41.7%)	0.211
Moderate (*n* = 62)	46	(74.2%)	16	(25.8%)	0.192^a^
Poor (*n* = 16)	11	(68.8%)	5	(31.2%)	
Tumor stage					
Early (I–II) (*n* = 48)	41	(85.4%)	7	(14.6%)	*0.001*
Advanced (III–IV) (*n* = 78)	44	(56.4%)	34	(43.6%)	
Skin invasion					
No (*n* = 96)	73	(76.0%)	23	(24.0%)	*<0.001*
Yes (*n* = 30)	12	(40.0%)	18	(60.0%)	
Nerve invasion					
No (*n* = 86)	63	(73.3%)	23	(26.7%)	*0.042*
Yes (*n* = 40)	22	(55.0%)	18	(45.0%)	
Blood vessel invasion					
No (*n* = 122)	83	(68.0%)	39	(32.0%)	0.595^b^
Yes (*n* = 4)	2	(50.0%)	2	(50.0%)	
Lymphatic invasion					
No (*n* = 122)	84	(68.9%)	38	(31.1%)	0.101^b^
Yes (*n* = 4)	1	(25.0%)	3	(75.0%)	
Tumor depth ≥10 mm					
No (*n* = 60)	52	(86.7%)	8	(13.3%)	*<0.001*
Yes (*n* = 66)	33	(50.0%)	33	(50.0%)	
Bone invasion					
No (*n* = 92)	68	(73.9%)	24	(26.1%)	*0.011*
Yes (*n* = 34)	17	(50.0%)	17	(50.0%)	

*ECS* extracapsular spread

^a^χ^2^ trend test

^b^Fisher’s exact test

In tongue cancer (Table 4), CRP elevation (CRP ≥ 5.0 mg/L) showed a strong relationship with advanced pathological tumor status (*p* < 0.001) and correlated with advanced pathologic nodal status (*p* = 0.027) and advanced tumor stage (*p* = 0.021).

**Table 4:** Association between CRP and clinicopathologic parameters in tongue cancer (*n* = 132)

Characteristic	CRP				*p* value
	Negative		Positive		
Pathologic tumor status					
Early (T1–T2) (*n* = 96)	83	(86.5%)	13	(13.5%)	*<0.001*
Advanced (T3–T4) (*n* = 36)	19	(52.8%)	17	(47.2%)	
Pathologic N stage					
N0 (*n* = 77)	64	(83.1%)	13	(16.9%)	*0.027*
N1 (*n* = 15)	13	(86.7%)	2	(13.3%)	*0.017* ^a^
N2 (*n* = 40)	25	(62.5%)	15	(37.5%)	
Nodal status					
(−) metastasis, (−) ECS (*n* = 77)	64	(83.1%)	13	(16.9%)	0.163
(+) metastasis, (−) ECS (*n* = 17)	12	(70.6%)	5	(29.4%)	0.066^a^
(+) metastasis, (+) ECS (*n* = 38)	26	(68.4%)	12	(31.6%)	
Differentiation					
Well (*n* = 33)	28	(84.8%)	5	(15.2%)	0.152
Moderate (*n* = 81)	63	(77.8%)	18	(22.2%)	
Poor (*n* = 18)	11	(61.1%)	7	(38.9%)	
Tumor stage					
Early (I–II) (*n* = 64)	55	(85.9%)	9	(14.1%)	*0.021*
Advanced (III–IV) (*n* = 68)	47	(69.1%)	21	(30.9%)	
Skin invasion					
No (*n* = 131)	101	(77.1%)	30	(22.9%)	1.000
Yes (*n* = 1)	1	(100.0%)	0	(0.0%)	
Nerve invasion					
No (*n* = 77)	60	(77.9%)	17	(22.1%)	0.833
Yes (*n* = 55)	42	(76.4%)	13	(23.6%)	
Blood vessel invasion					
No (*n* = 130)	100	(76.9%)	30	(23.1%)	1.000^b^
Yes (*n* = 2)	2	(100.0%)	0	(0.0%)	
Lymphatic invasion					
No (*n* = 129)	101	(78.3%)	28	(21.7%)	0.129^b^
Yes (*n* = 3)	1	(33.3%)	2	(66.7%)	
Tumor depth (≥10 mm)					
No (*n* = 58)	49	(84.5%)	9	(15.5%)	0.080
Yes (*n* = 74)	53	(71.6%)	21	(28.4%)	
Bone invasion					
No (*n* = 129)	100	(77.5%)	29	(22.5%)	0.542^b^
Yes (*n* = 3)	2	(66.7%)	1	(33.3%)	

*ECS* extracapsular spread

^a^χ^2^ trend test

^b^Fisher’s exact test

In other tumor subsites (Table 5), the CRP elevation (CRP ≥ 5.0 mg/L) was significantly associated with advanced pathological tumor status (*p* = 0.001), tumor stage (*p* = 0.009), and bone invasion (*p* < 0.001). It was also correlated with tumor depth ≥10 mm (*p* = 0.009).

**Table 5:** Association between CRP and clinicopathologic parameters in subsites other than tongue and buccal mucosa (*n* = 85)

Characteristic	CRP				*p* value
	Negative		Positive		
Pathologic tumor status					
Early (T1–T2) (*n* = 43)	38	(88.4%)	5	(11.6%)	*0.001*
Advanced (T3–T4) (*n* = 42)	24	(57.1%)	18	(42.9%)	
Pathologic N stage					
N0 (*n* = 54)	40	(74.1%)	14	(25.9%)	0.277
N1 (*n* = 14)	8	(57.1%)	6	(42.9%)	0.765^a^
N2 (*n* = 17)	14	(82.4%)	3	(17.6%)	
Nodal status					
(−) metastasis, (−) ECS (*n* = 54)	40	(74.1%)	14	(25.9%)	0.915
(+) metastasis, (−) ECS (*n* = 15)	11	(73.3%)	4	(26.7%)	0.694^a^
(+) metastasis, (+) ECS (*n* = 16)	11	(68.8%)	5	(31.2%)	
Differentiation					
Well (*n* = 26)	21	(80.8%)	5	(19.2%)	0.425
Moderate (*n* = 49)	35	(71.4%)	14	(28.6%)	0.195^a^
Poor (*n* = 10)	6	(60.0%)	4	(40.0%)	
Tumor stage					
Early (I–II) (*n* = 30)	27	(90.0%)	3	(10.0%)	*0.009*
Advanced (III–IV) (*n* = 55)	35	(63.6%)	20	(36.4%)	
Skin invasion					
No (*n* = 77)	57	(74.0%)	20	(26.0%)	0.677^a^
Yes (*n* = 8)	5	(62.5%)	3	(37.5%)	
Nerve invasion					
No (*n* = 66)	48	(72.7%)	18	(27.3%)	0.934
Yes (*n* = 19)	14	(73.7%)	5	(26.3%)	
Blood vessel invasion					
No (*n* = 79)	59	(74.7%)	20	(25.3%)	0.337^a^
Yes (*n* = 6)	3	(50.0%)	3	(50.0%)	
Lymphatic invasion					
No (*n* = 83)	60	(72.3%)	23	(27.7%)	0.383
Yes (*n* = 2)	2	(100.0%)	0	(0.0%)	
Tumor depth ≥10 mm					
No (*n* = 42)	36	(85.7%)	6	(14.3%)	*0.009*
Yes (*n* = 43)	26	(60.5%)	17	(39.5%)	
Bone invasion					
No (*n* = 54)	47	(87.0%)	7	(13.0%)	*<0.001*
Yes (*n* = 31)	15	(48.4%)	16	(51.6%)	

*ECS* extracapsular spread

^a^χ^2^ trend test

† Fisher’s exact test

### The association between CRP and survival

**Fig. 2:**
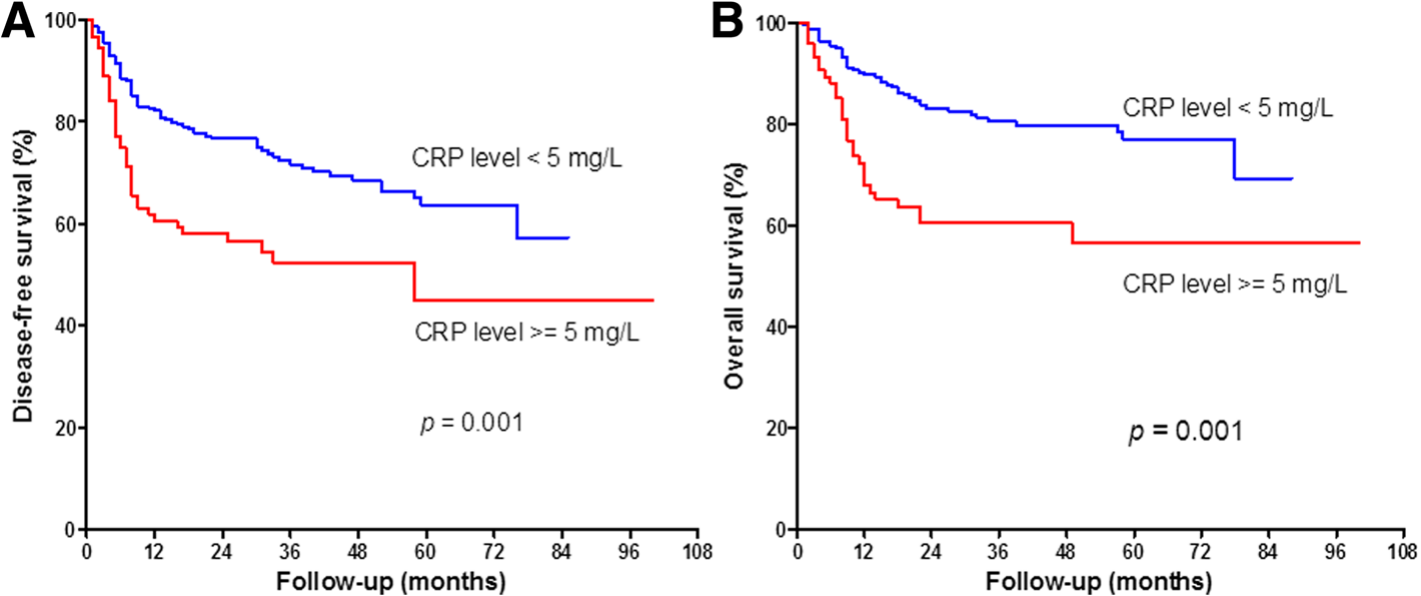
Survival curves in 343 OSCC patients related to the preoperative CRP level. **a** The lower CRP level group showed significantly better DFS compared to the higher CRP group (*p* < 0.001). **b** The lower CRP level group showed significantly better OS compared to the higher CRP level group (*p* < 0.001)

Comparing the prognosis between the two by univariate analysis, the group with lower CRP (CRP < 5.0 mg/L) has a longer disease-free survival (DFS) than the high CRP group (CRP ≥ 5.0 mg/L), (log-rank test *p* ≤ 0.001, Fig. 2a). Similarly, overall survival (OS) is longer in the low CRP level group (CRP < 5.0 mg/L) compared to the high CRP level group (CRP ≥ 5.0 mg/L) (log-rank test *p* ≤ 0.001, Fig. 2b). The hazard ratio (HR) for CRP including all subsites, DFS 1.902 (95% confidence interval (CI) 1.302–2.778) and OS 2.235 (95% CI 1.393–3.585). We analyzed the influence of CRP on survival according to different subsites: the HR for CRP in tongue cancer DFS 1.785 (95% CI 0.848–3.757); OS 1.535 (95% CI 0.630–3.741); the HR for CRP in buccal cancer, DFS 2.293 (95% CI 1.309–4.017); OS 3.610 (95% CI 1.732–7.526); the HR for CRP in other cancer subsites, DFS 1.577 (95% CI 0.721–3.449), OS 1.252 (95% CI 0.403–3.885).

## Discussion

Our previous studies showed a positive relationship between CRP level elevation and advanced oral cavity cancer stage [6]. Therefore, CRP has the potential to be a biomarker for oral cavity cancer and a predictor of prognosis before treatment. In this study, we recruited more cases (343 cases) to evaluate the connection between preoperative serum CRP level, oral cavity cancer stage, and prognosis. In the present study, oral cavity cancer had a greater prevalence between males, with mean age falling in the middle age period. This distribution was probably due to greater exposure to oral cavity cancer risk factors (smoking, drinking, and chewing betel nut habit) [14–17] in this subgroup. In contrary, the most common site of oral cavity cancer in the Western population is the tongue. However, in Taiwan, due to betel nut chewing, the common sites of oral cavity cancer are buccal mucosa and tongue, compatible with our patients’ tumor site distribution [18, 19].

**Fig. 3:**
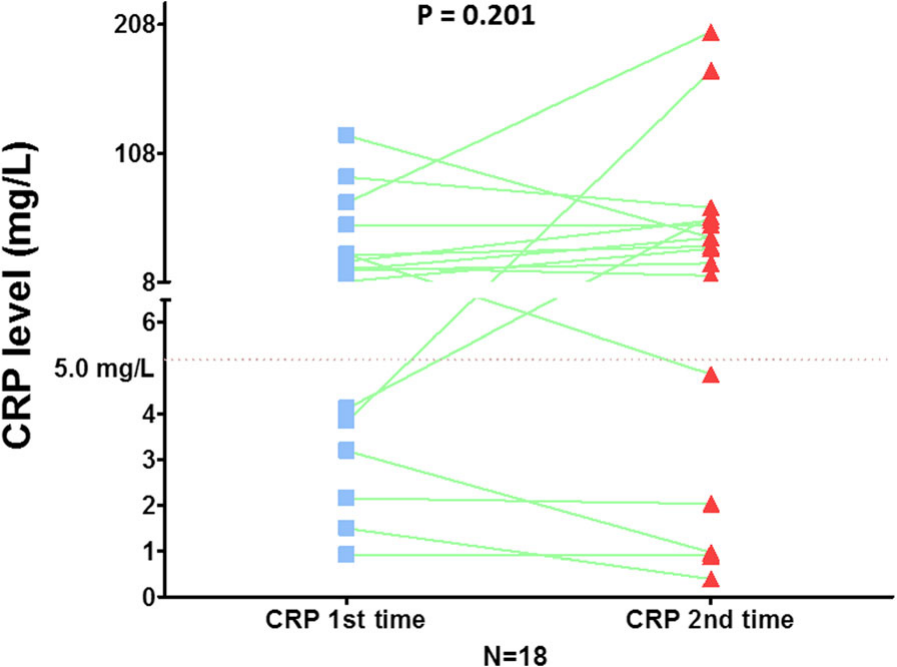
The change of preoperative serum CRP levels in 18 OSCC who had tests of CRP level twice before surgery

It is still a debate if the elevation of CRP could be due to a concomitant pulmonary infection or other infection, and be non-specific for oral cancer. In 18 of our cases, the CRP levels were checked twice. The first test was at the time of diagnosis, and the second test was performed the day before surgery. Eighty-three percent of the patients had similar levels or a more elevated CRP level in the second test; only three cases had lower CRP levels in the second test (Fig. 3, paired *t* test *p* = 0.201). This indicated that the serum CRP levels are stable in OSCC cancer patients, and so, the serum level analyzed in our study was not amenable to change in a different time period.

CRP is an acute phase protein as the host reacts to an inflammatory response and released from the liver into the bloodstream. Because of its short plasma half-life and robust reaction, it has been used clinically as a marker for inflammatory or infectious status [10, 11, 20, 21]. Recently, CRP has proved a predictive factor in certain human cancers such as gastrointestinal, breast, lung, and gynecologic cancers [9, 22–26]. There are three mechanisms about the relationship between CRP elevation and cancer prognosis: (1) oxidative damage caused by inflammation promotes tumor growth, (2) the tumor growth and apoptosis induced the release of CRP, (3) inflammation is a contributing factor to tumor progression and reflects in the elevation of CRP. We demonstrated that the CRP level was closely related with an increased squamous cell carcinoma antigen (SCC-Ag) and neutrophil to lymphocyte ratio (NLR) in peripheral blood tests at the time of diagnosis in OSCC patients [7, 27]. The SCC-Ag was closely related with primary tumor status and lymph node metastasis, which could stand for the tumor burden [7, 28, 29]. The elevated neutrophil ratio could be from tumor growth and the consequent immune response from the host. Circulating neutrophils contain and secrete various cytokines including circulating matrix metalloproteinases [30], vascular endothelial growth factor (VEGF) [31], platelet-derived growth factor, fibroblast growth factor, CXCL8 [32], elastases [33], and interleukin-8 [34]. These cytokines create a microenvironment that facilitates extracellular matrix remodeling, endothelial cell migration, and tumor cell invasion. Also, the released cytokines such as IL-6 could further stimulate the production of CRP in the liver [8].

In this study, CRP elevation and tumor status presented a clear positive correlation. To clarify the role of preoperative serum CRP level in OSCC, we stratified our patients into different subsites. There was a similar relationship between CRP elevation and tumor status for each cancer subsite. In the literature, nine studies investigated the roles of CRP in OSCC (Table 6) [6, 35–40]. Only one study did not find any association between CRP elevation and survival. Another point to consider is the fact that the included studies used inconsistent cut-off values for CRP levels, which caused heterogeneity in the influence of CPR in prognosis. In a meta-analysis in urologic malignancies, a similar condition of different cut-off points was observed, and they found setting the level at 5.0 mg/L was the most appropriate [41].

**Table 6:** Literature review for the relationship of CRP level and survival in OSCC patients

Authors/year	Tumor sites/nations	Case no.	Cut-off point, tumor stage	Disease-free survival (DFS)	Overall survival (OS)
Jablonska E. et al. (1997) [5]	OSCC/Poland	42	Compares mean values, significant difference between stages IV vs II (*p* < 0.01)	NA	NA
Khandavilli S.D. et al. (2009) [36]	OSCC/United Kingdom	60	CRP > 5.0 mg/L; significant with tumor stage, *p* = 0.046	NA	Significant in OS (*p* < 0.001) (multivariate)
Kruse A.L. et al. (2010) [37]	OSCC/Switzerland	278	CRP > 5.0 mg/L; NA	No significant difference (*p* = 0.137)	Not significant in time to metastasis (*p* = 0.468)
Chen, H.H. et al. (2011) [6]	OSCC/Taiwan	59	CRP ≥ 5.0 mg/L, significant with tumor stage, *p* = 0.005	Significant difference in 2-year DFS, *p* < 0.001	Significant difference in 2-year OS, *p* = 0.013
Grimm, M. et al. (2012) [45]	OSCC/Germany	187	CRP ≥ 1.1 mg/L; LPI significantly correlated with advanced tumor stage, *p* = 0.0002^a^	5-year DFS, *p* < 0.0001	NA
Huang SF et al. (2012) [7]	OSCC/Taiwan	142	CRP ≥ 5.0 mg/L, significantly related with tumor stage, *p* = 0.001	3-year DFS, *p* < 0.001	3-year OS, *p* = 0.008
Peter F. et al. (2013) [38]	HNSCC/Germany	261	CRP ≥ 2.0 mg/L, significant with advanced tumor stage, *p* = 0.006; nodal status, *p* = 0.045	NA	5-year OS, *p* < 0.0001
Chen, I.H. et al. (2014) [39]	OSCC/Taiwan	Recurrent OSCC, 100	CRP ≥ 5.0 mg/L, significant with tumor stage, *p* < 0.001	NA	Mean 33.53 months, OS significantly different, *p* < 0.001
Farhan-Alanie, O.M. et al. (2015) [40]	OSCC and soft palatal ca/ United Kingdom	178	Modified Glasgow scale: combined albumin (<35 g/L) and CRP (>10 mg/L) level	Modified Glasgow scale related with cancer-specific survival (*p* < 0.001)	Modified Glasgow scale related with overall survival (*p* < 0.001)

^a^Combines CRP, Hb, and WBC as laboratory prognostic index (LPI)

In this study, we found an elevated CRP was highly correlated with primary tumor status, tumor depth, and lymph node metastasis in OSCCs. Regarding the tumor subsites, the elevation of CRP in buccal cancer had the most promising association with most clinicopathologic factors. In addition, the elevation of CRP was related with lymph node metastasis in buccal and tongue cancers, while the association between increased CRP and ECS was only found in buccal cancer (Tables 3, 4, and 5). The stronger relationship between CRP and buccal cancer in our series may be due the high incidence of AQ consumption in our population, making the buccal mucosa the site of greatest risk of contracting malignancy in betel quid chewers [42, 43]. The intimate contact between the buccal mucosa and the AQ during chewing induces chronic and abnormal mucosa inflammation by promoting the release of inflammatory mediators like IL-6, TNF-α, and PGE_2_ by oral keratinocytes [44], playing a crucial role in the pathogenesis of oral cancer.

We believe that there are limitations in this study. The major one comes from the case number in each tumor subsite. However, from this preliminary analysis, we believe that determining CRP levels preoperatively, especially in buccal cancer, would be relevant and useful to clinicians because CRP measurement is rapid, inexpensive, and repeatable in a clinical setting. Using CRP as a biomarker could help clinicians select proper treatment strategies for patients with OSCC, detecting which patients would benefit from adjuvant treatment by predicting pathologically aggressive tumors based on their CRP levels [6].

## Conclusions

The presence of an elevated serum CRP level preoperatively (≥5.0 mg/L) is an important prognostic indicator in oral cancer in the Taiwanese population. Elevated CRP levels are associated with tumor stage and locoregional invasiveness. Furthermore, the prognostic prediction is more evident in buccal cancer, which could be attributed to the tumor’s behaviors related with AQ and tobacco use.

## Additional files

Additional file 1: Clinical information of 343 OSCC patients. (SAV 100 kb)

## Abbreviations

CI: Confidence interval
CRP: C-reactive protein
DFS: Disease-free survival
ECS: Extracapsular spread
HR: Hazard ratio
NLR: Neutrophil to lymphocyte ratio
OS: Overall survival
OSCC: Oral cavity squamous cell carcinoma
PET: Positron emission tomography
SCC-Ag: Squamous cell carcinoma antigen
SD: Standard deviation
VEGF: Vascular endothelial growth factor
